# Case Report: Five positive lesions on bilateral adrenal glands detected by a single ^68^Ga-pentixafor PET/CT

**DOI:** 10.3389/fnume.2026.1676221

**Published:** 2026-04-10

**Authors:** Ze Mao, Lu Zheng, Ranliang Hua, Detao Wu, Yaai Shi, Xinchao Zhang, Yujing Hu, Yanzhu Bian

**Affiliations:** 1 Department of Nuclear Medicine, Hebei General Hospital, Shijiazhuang, Hebei, China; 2 Department of Emergency, Hebei General Hospital, Shijiazhuang, Hebei, China

**Keywords:** ^68^Ga-Pentixafor, aldosterone-producing adenoma, CXCR4, PET/CT, primary aldosteronism

## Abstract

**Background:**

Primary aldosteronism (PA) is an endocrine disorder caused by autonomous aldosterone hypersecretion from the adrenal zona glomerulosa, causing sodium retention, plasma volume expansion, and suppressed renin activity, manifesting as hypertension with/without hypokalemia. Aldosterone-producing adenomas (APAs), a common PA subtype, are typically unilateral and solitary; bilateral involvement is rare. We report a patient with hypertension and bilateral multiple adrenal adenomas. ^68^Ga-Pentixafor PET/CT proved crucial for detecting and subtyping lesions, highlighting its significant diagnostic utility for APA subtyping.

**Case presentation:**

A 62-year-old male presenting with nausea, generalized fatigue, and poorly controlled hypertension (10-year history; max BP 200/110 mmHg) was admitted. Bilateral adrenal CT, which revealed multiple bilateral adrenal adenomas, raised clinical suspicion for primary aldosteronism. To confirm the diagnosis, ^68^Ga-Pentixafor PET/CT imaging was performed, which identified five nodules with varying intensities of tracer uptake bilaterally.

**Conclusion:**

^68^Ga-Pentixafor PET/CT integrates structural and functional assessment, transcending the limitations of CT and adrenal vein sampling (AVS), thereby demonstrating substantial clinical value for evaluating multifocal or bilateral adrenal pathologies.

## Introduction

Aldosterone-producing adenomas (APAs) represent a predominant subtype of primary aldosteronism (PA), characterized by hypokalemia, elevated aldosterone levels, and Aldosterone to Renin Ratio (ARR) ≥ 30 (1). Precise subtype diagnosis is paramount for clinical management. Currently, APA diagnosis relies heavily on adrenal CT and adrenal vein sampling (AVS) for lesion localization and functional assessment. However, CT frequently misses adenomas <1 cm in diameter and lacks functional characterization, while AVS is an invasive and technically challenging procedure, limiting its widespread application (2). C-X-C chemokine receptor type 4 (CXCR4), a G-protein-coupled receptor (GPCR), is expressed on the cell membrane of APA cells (3). ^68^Ga-Pentixafor, a high-affinity ligand for CXCR4, enables specific *in vivo* targeting. This allows CXCR4-directed PET/CT functional imaging, providing significant value for PA subtyping and guiding clinical treatment decisions. We report a case where a single ^68^Ga-Pentixafor PET/CT scan identified five positive lesions on bilateral adrenal glands, highlighting its diagnostic impact for precise localization and functional characterization in the context of bilateral multiple adrenal adenomas.

## Case presentation

**Figure 1:**
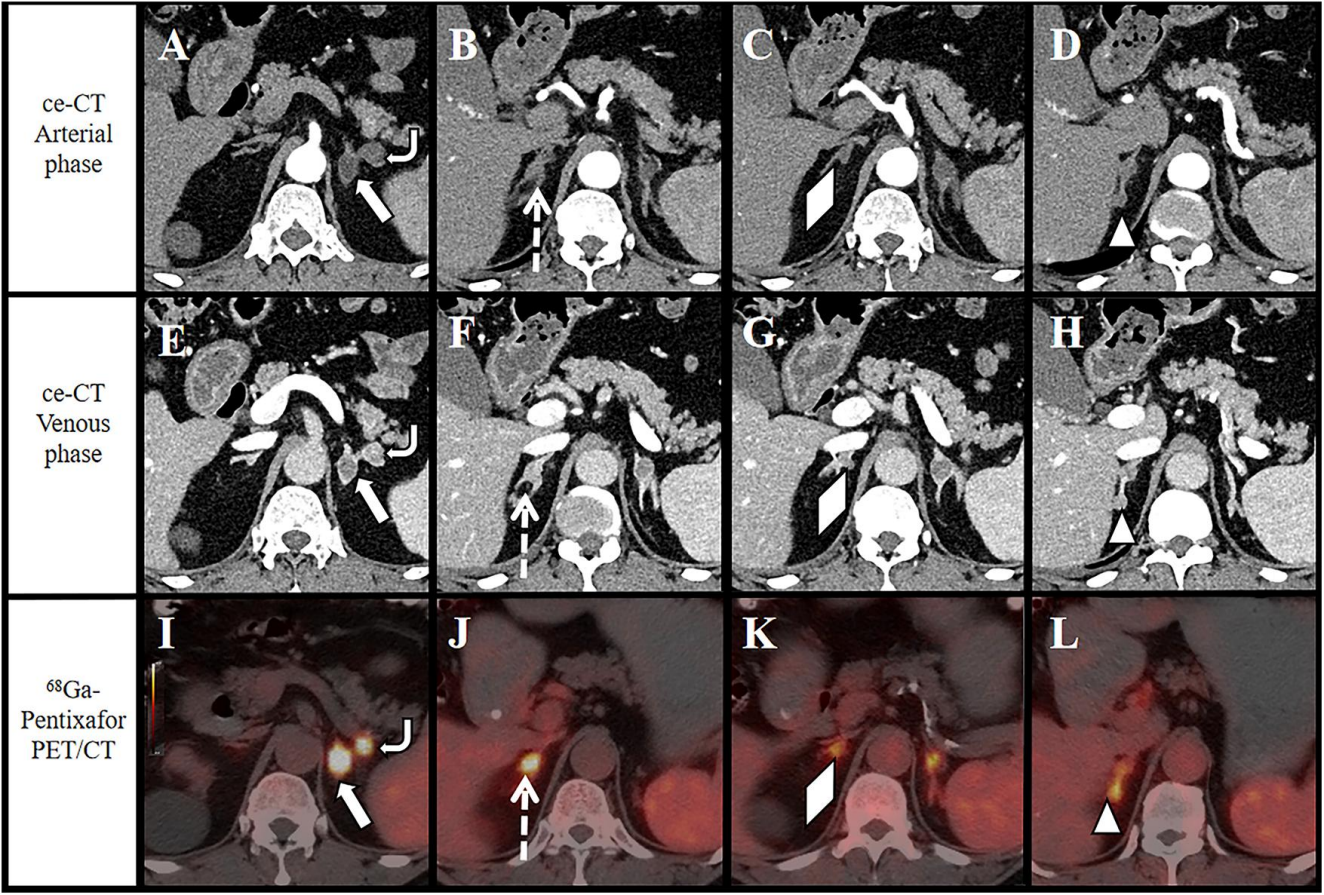
Three well-visualized adenomas are demonstrated on CT imaging. Contrast-enhanced(ce) CT arterial phase **(A)** and venous phase **(E)** revealed a quasi-spherical nodule in the left adrenal gland [17 mm; Hounsfield unit (HU) = 8.23; arrow] and a nodule in its lateral limb (19 mm × 14 mm; HU = 3.67; rounded arrow); ce CT arterial phase **(B)**, and venous phase **(F)** showed an irregular nodule in the right adrenal body (16 mm × 11 mm; HU = 19.96, dashed arrow); all the 3 nodules exhibiting moderate enhancement with a rapid “fast-in-fast-out” pattern during arterial and venous phases. Fusion images from ^68^Ga-pentixafor PET/CT demonstrated significant radiotracer uptake in the bilateral adrenal. Three adenomas identified on CT demonstrated avid uptake on ^68^Ga-pentixafor PET/CT: **(I)**, (arrow; SUVmax 31.30;rounded arrow; SUVmax 15.57); **(J)**, (dashed arrow; SUVmax 7.50). Two metabolically active lesions were newly detected in the right adrenal gland and its medial branch: **K**, (triangular arrow; SUVmax 5.43; 10 × 9 mm; HU 19.21); **(L)**, (diamond-shaped arrow; SUVmax 5.38; 10 × 7 mm; HU -1.69).

A 62-year-old male was admitted with nausea, vomiting, and generalized fatigue. He had a 10-year history of hypertension with a maximum recorded blood pressure of 200/110 mmHg. Laboratory findings revealed hypokalemia (serum potassium: 2.5 mmol/L), elevated plasma aldosterone concentration (PAC) [upright Renin-Angiotensin-Aldosterone System (RAAS) test: 771.100 pg/mL], ARR > 37, and a positive captopril suppression test (aldosterone pre-captopril: 1036.200 pg/mL; post-captopril: 1013.300 pg/mL). On contrast-enhanced CT, all lesions exhibited mild-to-moderate heterogeneous enhancement with a rapid wash-in and wash-out pattern (Figures 1A–H). The clinical team suspicion for primary aldosteronism arose, but AVS failed to lateralize the side of dominant aldosterone secretion. Consequently, ^68^Ga-Pentixafor PET/CT (Figure 1) was performed for further subtyping.

PET/CT imaging was performed 30 min after intravenous injection of 3.5 mCi (129.5 MBq) of ^68^Ga-Pentixafor by a nuclear medicine physician. PET/CT images demonstrate five distinct nodular lesions in the bilateral adrenal glands, with varying degrees of tracer uptake in all five nodules (Figures 1I–L). Among the five adrenal nodules, the nodule in the medial branch of the left adrenal gland exhibited the highest metabolic activity, with a maximum standardized uptake value (SUVmax) of 31.3. The mean SUV (SUVmean) of normal liver parenchyma was 2.26. The lesion-to-liver ratios (LLR; calculated as SUVmax lesion/SUVmean liver) for the adrenal lesions ranged from 2.38 to 13.85. The SUVmean of normal adrenal cortex was 3.41. The lesion-to-normal-adrenal ratios (LAR; SUVmax lesion/SUVmean normal adrenal) ranged from 1.58 to 9.18. In ⁶⁸Ga-Pentixafor PET/CT imaging, the thresholds for diagnosing APA are defined as LLR≥2.5 and LAR≥2.4 (4). In this case, ⁶⁸Ga-Pentixafor PET/CT detected five adrenal adenomas, all of which exhibited heterogeneous tracer uptake with LLR and LAR values both exceeding the aforementioned diagnostic thresholds.^68^Ga-Pentixafor PET/CT identified five adrenal adenomas demonstrating heterogeneous tracer uptake. The clinical team integrating these findings with the laboratory results and contrast-enhanced CT, all five adenomas were considered functional APAs. Therefore, conservative medical management was initiated. After switching the antihypertensive regimen to a mineralocorticoid receptor antagonist for 30 days, the patient's serum potassium normalized to 4.3 mmol/L and blood pressure remained within normal limits.

## Discussion

PA is an endocrine disorder characterized by autonomous, excessive aldosterone secretion from the zona glomerulosa of the adrenal cortex. This leads to sodium retention and plasma volume expansion, consequently suppressing renin activity. Clinical manifestations typically include hypertension with or without hypokalemia, while a minority of patients may experience paresthesias or carpopedal spasm (5). The two principal subtypes are APA and idiopathic hyperaldosteronism (IHA), making subtype differentiation a critical aspect of clinical management (5). APAs typically present as unilateral, solitary adrenal lesions, with simultaneous bilateral APA involvement being uncommon.

Laboratory findings in APA often include hypokalemia (serum K^+^ < 3.5 mmol/L).PAC is typically elevated. During upright RAAS testing, normal upright PAC levels range from 70 to 300 pg/mL. The ARR is widely used as a screening tool; an ARR ≥ 30 strongly suggests PA. Furthermore, a positive captopril challenge test (defined as <30% decrease or an increase in PAC post-captopril) supports an APA diagnosis.

Current APA diagnosis primarily relies on adrenal imaging and AVS for lesion localization and functional assessment (6, 7). Computed tomography (CT), as the first-line imaging modality, typically reveals unilateral or bilateral adrenal adenomas (<2 cm in diameter), appearing oval/round, well-circumscribed, with ring-like enhancement on contrast study and a hypodense center, without ipsilateral or contralateral adrenal atrophy. However, CT has limitations: it may miss smaller adenomas (short axis <1 cm) and cannot differentiate functional status (5, 8). AVS is considered the current diagnostic ‘gold standard' for identifying the source (unilateral vs. bilateral) of aldosterone excess, establishing lateralization which is crucial for treatment selection. Nevertheless, AVS is an invasive, technically challenging procedure associated with complication risks, hindering its widespread implementation (9, 10). Furthermore, APAs result in excessive aldosterone secretion on both sides, thereby imposing certain limitations on AVS application and potentially obscuring the identification of the dominant side (11).

C-X-C chemokine receptor type 4 (CXCR4), a G-protein-coupled receptor, is highly expressed on APA cell membranes and strongly correlates with aldosterone synthase (CYP11B2) expression (12–14). However, the expression of this receptor is not specific to APAs and is also detectable in other adrenal lesions such as cortisol-producing adenomas (15). ^68^Ga-Pentixafor specifically targets membrane CXCR4. This functional PET/CT imaging modality aids PA subtyping and clinical decision-making (4, 15). Studies indicate that functional APAs show significantly higher ^68^Ga-Pentixafor uptake compared to nonfunctional adrenal nodules on the contralateral side, with over 90% of functional nodules demonstrating tracer uptake (12).

In this case, ^68^Ga-Pentixafor PET/CT identified five adrenal adenomas exhibiting heterogeneous tracer uptake. Correlated with laboratory findings and contrast-enhanced CT, these were diagnosed as APAs. For patients with bilateral tracer-avid lesions on ^68^Ga-Pentixafor PET/CT, treatment strategy should be individualized based on lesion size, number, and SUVmax values to assess the relative dominance on each side.

Current APA treatments include adrenalectomy(surgical removal of the dominant aldosterone-secreting gland) and medical therapy. However, non-dominant lesions on the contralateral side may still possess autonomous aldosterone secretion. If hypertension or hypokalemia persists post-operatively or recurs, further medical therapy or repeat surgery may be required. For patients unsuitable for or declining surgery, mineralocorticoid receptor antagonists (MRAs) are the mainstay medical treatment (16, 17). Our patient opted for medical management, achieving significant improvement in both hypokalemia and hypertension during follow-up. Furthermore, the SUVmax of the lesion in the medial limb of the left adrenal gland was markedly higher than those of the remaining lesions in this case, suggesting a potential positive correlation between SUVmax and aldosterone secretion levels. Ding (3) confirmed that CXCR4 was significantly positively correlated with CYP11B2, and SUVmax was also significantly positively correlated with CXCR4 expression. These findings further indicate that SUVmax can indirectly reflect the aldosterone-secreting capacity of lesions, though this conclusion still requires validation in larger sample sizes.

^68^Ga-Pentixafor could enables molecular characterizations of CXCR4 in PA lesions; thus,^68^Ga-Pentixafor PET/CT holds significant clinical value in APA subtyping diagnosis, determining the functional lateralization of aldosterone-producing lesions, and guiding treatment decisions. As a non-invasive technique providing simultaneous morphological and functional assessment, ^68^Ga-Pentixafor PET/CT compensates for the limitations of CT—its inability to characterize the functional status of lesions—and the technical challenges associated with performing AVS in this case. Thus, it holds considerable clinical value for the localization and functional characterization of bilateral adrenal adenomas. However, as this study is a single case report with a limited sample size, the clinical value of its conclusions still requires further validation.

## Conclusions

^68^Ga-Pentixafor PET/CT identified five positive lesions on bilateral adrenal glands, the clinical team confirming bilateral multifocal APA. Given the pattern of bilateral autonomous secretion without unilateral dominance, medical therapy with aldosterone antagonists is the primary treatment. Surgery is unsuitable for such diffuse bilateral involvement. ^68^Ga-Pentixafor PET/CT enables optimal subtype differentiation and treatment guidance.

## Data Availability

The original contributions presented in the study are included in the article/Supplementary Material, further inquiries can be directed to the corresponding authors.
